# Adjustment of the data acquisition window for the assessment of sensorimotor gating mechanisms in rodents

**DOI:** 10.1016/j.mex.2019.09.007

**Published:** 2019-09-12

**Authors:** Sebastian Hormigo, Dolores E. López

**Affiliations:** a Institute for Neuroscience of Castilla y León (INCYL), University of Salamanca, Salamanca, Spain; b Institute of Biomedical Research of Salamanca (IBSAL), University of Salamanca, Salamanca, Spain; c Department of Cell Biology and Pathology, University of Salamanca, Salamanca, Spain

**Keywords:** Adjustment of the response window for measurement of ASR, Acoustic startle reflex, Data acquisition window, Fast Fourier transform, Response window, Sampling, Sensorimotor gating

## Abstract

The acoustic startle reflex (ASR) is a short and intense defensive reaction in response to a loud and unexpected acoustic stimulus. The ASR can be modulated through sensorimotor gating processes, such as prepulse inhibition (PPI), a neurological phenomenon in which a weak pre-stimulus inhibits the reaction to a startling stimulus. The reduction of the amplitude of ASR reflects the ability of the CNS to adapt to a salient sensory stimulus when a preceding weaker signal is perceived. Despite its obvious importance for translational research studies, when the data acquisition window is not properly configured, the measurement of ASR may contain artifacts or be incorrect altogether. In this paper, we issue our recommendations for the correct definition of the response window to achieve good-quality ASR/PPI measurements in order to standardize and implement the method across conditions.

• Parameters for detection of peak responses need to be carefully configured, otherwise the risk of obtaining unwanted artifacts or high signal-to-noise ratio increases considerably.
• Custom settings yield traces with tighter baseline, higher amplitude, and shorter latency compared to factory default settings.
• FFT heatmaps show a solid color-correlation when using custom settings, without the appearance of artifacts.

## Graphical abstract

**Figure:**
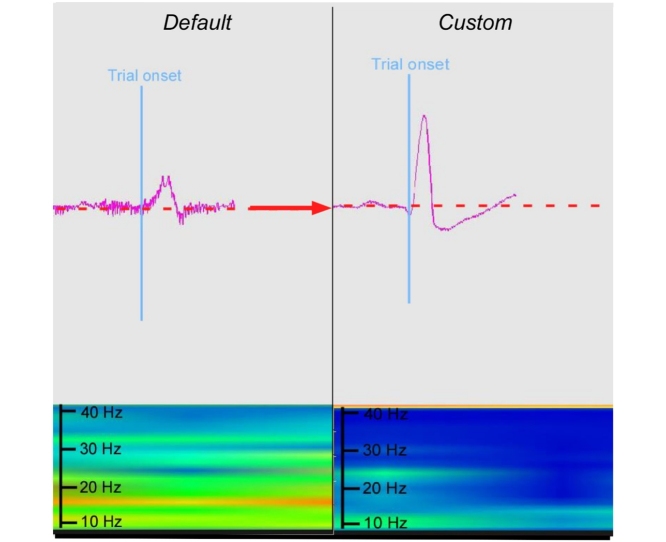

**Specification Table**

**Table:** 

Subject Area:	Neuroscience
More specific subject area:	Behavioral and cognitive neuroscience.
Method name:	Adjustment of the response window for measurement of ASR
Name and reference of original method:	N/A
Resource availability:	N/A

## Method details

### Background

The acoustic startle reflex (ASR) is a short and intense motor reaction that involves contraction of large numbers of muscle groups throughout the body in response to a loud and unexpected acoustic stimulus. The ASR serves as a defensive reflex triggered by the brainstem against possible aggression or alert against unexpected events. Indeed, the primary ASR pathway involves just three synapses in the rat central nervous system: onto the cochlear root neurons, the pontine reticular formation, and the spinal motoneurons [1].

The ASR can be modulated quantitatively or qualitatively through sensorimotor gating processes by a series of natural or experimental conditions. One of the more studied modulations of ASR is prepulse inhibition (PPI), a neurological phenomenon in which a weaker pre-stimulus (prepulse) inhibits the reaction to a subsequent strong startling stimulus (pulse). The PPI is an adaptive mechanism to prevent over-stimulation, helping the brain focus on a specific stimulus among other distracters [2]. Consequently, PPI provides operational measures of information processing by filtering irrelevant stimuli. Since ASR and PPI can be easily tested in humans and rats, these behavioral paradigms are used as valuable tools for translational research on disorders with attentional and information-processing dysfunctions [3]. The modulations of the ASR are related to structures of the central nervous system that act on some of the descending systems and end up influencing the activity of the nuclei in the primary ASR pathway.

In a recent work by Hormigo et al. [4], we studied the effects of cholinesterase reversible inhibitor *rivastigmine* on sensorimotor gating, memory, and learning tasks in healthy rats. As part of the sensorimotor gating assessment, we measured ASR and PPI. The section 2.3 of the above paper reads as follows:

### Acoustic startle reflex assessment

Using the SR-LAB system (SDI, San Diego, CA, USA), we assessed ASR parameters in a session routinely performed in our laboratory [[5], [6], [7], [8]]. Rats were exposed to a background white noise (65 dB SPL) that continued throughout the experimental session. A session consisted of an acclimatizing period of ∼5 min followed by 80 trials presented pseudo-randomly, with a mean inter-trial interval of 30 s. Sixteen of the trials involved a single-noise startling stimulus pulse (115 dB SPL, 20 ms burst of white noise). The remaining trials corresponded to 4 blocks of 16 trials consisting of a white-noise prepulse (80 dB SPL, 20 ms burst of white noise), followed by the startling stimulus (as described above). Each block comprised a different inter-stimulus interval (ISI): either 25, 50, 100, or 150 ms. Whole-ballistic movements of the rats corresponding to ASR were recorded with a piezoelectric accelerometer, converted from analog to digital signals, and analyzed by the SR-LAB software (version number 6500-0091-L) that provided two main values of interest: amplitude and latency. ASR amplitude represents the peak startle response (in arbitrary units) that occurs during each trial, while ASR latency is the time from stimulus onset to the peak startle response (in ms). The percentage of prepulse inhibition of the animal’s response to the startling stimuli was calculated for each respective ISI block according to the following formula: *%PPI* = *[(startle amplitude on pulse-alone trial* *−* *startle amplitude on prepulse to pulse trial (prepulses at 25, 50, 100, or 150 ms of ISI)/startle amplitude on pulse-alone trial]* *X* *100*. Prior to the first PPI testing session, all rats were handled and habituated to the startle stimulus a routinely performed in our lab.

In this current paper, we issue our recommendations for the definition of the response window (or data acquisition window) to achieve good-quality ASR/PPI measurements in order to standardize and implement the method across different experiments/experimenters. We also provide an example of a given signal acquired using our custom recommendations versus the factory default parameters, and the differences obtained.

### Adjustment of the response window (or data acquisition window)

*Base Line.* Average of the number of samples for the baseline calculation. It is measured in *ms*. ∞ values. Set to 5. [Factory settings: 1].

*Analysis Start.* The program searches for the start after this specification. Latencies are measured using this parameter. It is quantified in *ms*. Set to 0. [Factory settings: 5].

*End Analysis.* The program ignores data after this specification. Latencies are measured using this parameter. It is quantified in *ms*. Set to 250. [Factory settings: 500].

*Response Window.* It is the minimum time after the start of the trial for the peak detection. This forces the program to ignore a peak response due to something else rather than the stimulus. It is measured in *ms*. Ranges 5–100. Set to 5. [Factory settings: 100].

*Rolling Average.* It is the number of samples in the process of smoothness. Setting this parameter to 1 disables the function. Ranges 1–50. Set to 5. [Factory settings: 1].

*Filter Order.* It determines the sharpness of the Butterworth frequency digital filter. Setting this parameter to 0 disables the function. Ranges 1–50. Set to 2. [Factory settings: 0].

*Pass Frequency.* It configures the low-pass filter. It is set in Hz. Ranges 10–499. The higher this parameter is set, the better. But 250 should be enough. [Factory settings: 10].

*Response Criterion.* Multiple standard deviations of the amplitude above the baseline for the program to consider a peak response as valid. This parameter forces the program to ignore response peaks that do not have a significant amplitude, such as random animal movement inside the tube. Ranges 2–10. Set to 10. [Factory settings: 2].

*Record Samples.* This parameter asks for the number of samples to take in the recording of data. Any number can be introduced here. We suggest, at a minimum, introducing 1000 samples to obtain a good-quality recording. [Factory settings: 100].

*Samples per Second.* This parameter asks for the rate of sample recording (up to 2000). We suggest, at a minimum, introducing 1000 samples to obtain an adequate quality recording. Consider that *Record Samples* and *Samples per Second* in combination set the total duration of the recording for the response window. For example, 100 samples at a 1000 samples/second (1 per ms) yields a recording window of 100 ms. The window starts when the command *Record Data* is introduced in the definition of each trial. [Factory settings: 1000].

The above parameters must be set to satisfy the *Nyquist‐Shannon Theorem*, formulated for the first time by Harry Nyquist in 1928 ("Certain topics in telegraph transmission theory") [9], and formally demonstrated by Claude Shannon in 1949 ("Communication in the presence of noise") [10]. This theorem establishes a sufficient condition for a *sample rate* that permits discrete sequence of samples to capture all the information from a continuous signal of finite bandwidth. The theorem applies to a class of mathematical functions having a Fourier transform that is zero outside of a finite region of frequencies.

Briefly, regarding how the analysis work in the system (as stated in the system’s user manual):

“View Wave™” – This post session data analysis tool allows you to fully verify startle response data. The complete waveform can be reviewed for every response to verify the calculated numeric data. With the use of the Programmable Scoring Parameters, you can refine data via settable parameters that include Baseline, Onset Window Start, End Analysis and Onset criterion. Response Data Options – The SR-LAB software applications offer greater insight into the animal’s response to the startle stimulus. The expanded data response options included with SR-LAB are “Start, Baseline, Onset time to first peak, Time to maximum peak, Amplitude of first peak, Amplitude of maximum peak, and Average.”

Data Consolidation – SR-LAB software allows you to combine data from multiple startle sessions into a single Excel spreadsheet, into ASCII file format, or your choice of statistical programs. Just ‘point and click’ on the files to be consolidated and SR-LAB will preview the data and finish the process. The merged data can be sorted by Subject, Group, or ID.

Channel Consolidation – SR-LAB software allows you to define the stations to be run, the channels to be displayed and the subject information you want to include (i.e. Subject, Group and ID) in one Specification Window. Channel specifications can be saved for quick re-use in subsequent high throughput experiments.

More information available at https://sandiegoinstruments.com/product/sr-lab-startle-response/.

## Method validation

The Nyquist‐Shannon Theorem needs to be considered to adjust the ASR/PPI measurement system. This theorem refers to the process of data collection, not to be confused or associated with data quantification -the latter being a process that follows the sampling in the digitalization of a signal, and contrary to sampling, it is not reversible-. A loss of information in the sampling process, even under ideal circumstances, translates into a distortion known as quantification noise error. This establishes a theoretical upper limit to the signal-to-noise ratio. In other words, from the point of view of the Nyquist‐Shannon Theorem, discrete samples from a given signal are exact values that have not gone through rounding or clipping over a determined precision, thus not having been quantified yet. This theorem demonstrates the exact reconstruction of a continuous periodic signal from its samples is mathematically feasible if the signal is limited in its band and the sampling rate is over twice its bandwidth. In other words, the complete information of the original analog signal that complies with the above criterion is described by the total series of samples that resulted from the sampling process. Therefore, there is nothing in the development of the signal between samples that is not perfectly defined by the total series of samples.

For signals limited in band (or bandlimited), the Nyquist‐Shannon Theorem can be enunciated in 2 ways:

-
  - A signal limited in band that has no frequency components above W Hz can be represented in an exact form by specifying the signal values in time instants separated by Ts = 1/2 W seconds.
  - A signal limited in band that has no frequency components above W Hz can be represented in an exact form by a sampling rate fs = 2 W samples/second.

**Fig. 1:**
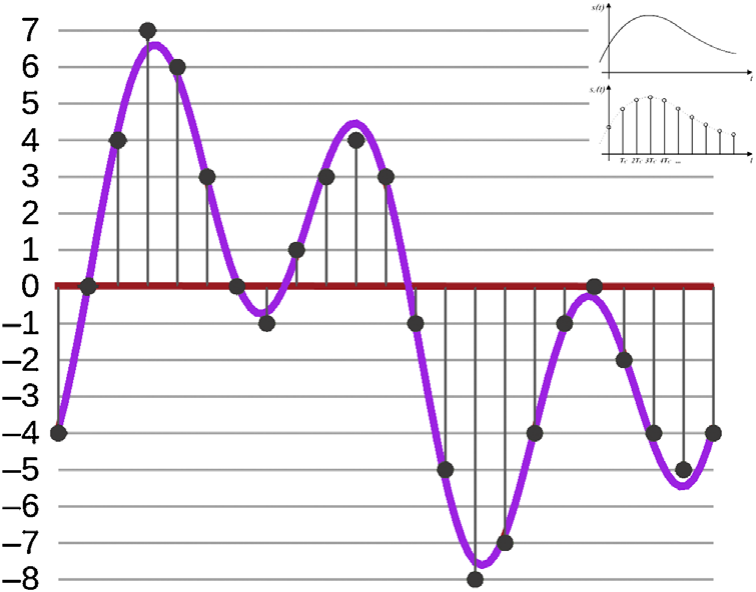
Sampling and reconstruction following the Nyquist‐Shannon Theorem. Example of a given trace sampled at a double-the-bandwidth rate to satisfy the theorem capturing all the information from a continuous signal. Right insets represent a continuous analog signal (top) and the sampled signal (bottom).

Therefore, the sampling frequency must be equal or above to twice the bandwidth of the analog signal for the Theorem to be satisfied (Fig. 1).

**Fig. 2:**
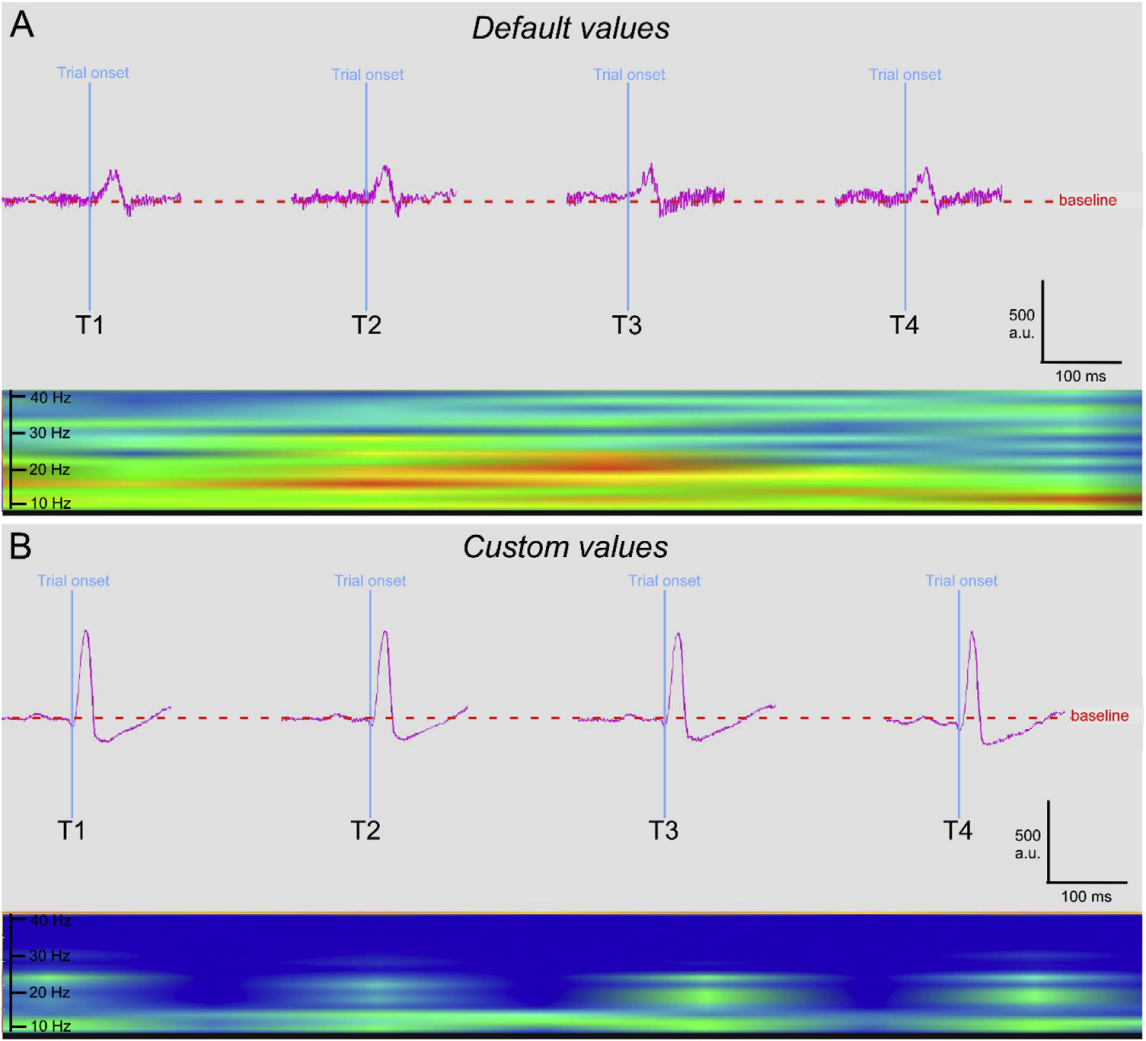
Validation of the custom data acquisition window. In A, four trials (T1–T4) acquired using the default factory settings. In B, four trials (T1–T4) acquired using our custom settings. Each panel (A and B) presents the traces for all the trials and a Fast Fourier Transform (FFT) heatmap visualization. Note the difference in trials: in A, baseline is noisy, amplitude is low, latency is long, and the FFT heatmap shows an increase in the mid-to-low frequency spectrum (ranging ∼20–10 Hz) and the appearance of high frequencies as well (∼35 Hz); whereas in B, baseline is tight, amplitude is high, latency is short, and the FFT heatmap shows a tighter color-correlation to the trace, without the appearance of non-desired frequencies (dark blue).

As an example of the difference in detected responses obtained using different parameters, we present the data yielded using the factory settings (Fig. 2A) compared to our custom settings (Fig. 2B). Factory default settings yielded traces that have a thick baseline, a byproduct from the pickup of high-frequency noise that has not been filtered (Fig. 2A). This is not present in the traces yielded from our custom settings, whose trace line is slim and accurate, having been filtered out of unwanted high-frequency noise (Fig. 2B).

Other notable differences between settings is the amplitude and latency of the traces. The factory settings yielded a peak detection around half the amplitude and double the latency of the peak yielded by the custom settings (Fig. 2A and B). The reason is a combination of the parameters *Analysis Start*, *End Analysis*, *Response Window*, and *Response Criterion* not being tuned-up for optimum performance. Factory settings detected a response that was out-of-phase compared to the real response, masked by the animal’s random movement inside the testing tube, and having the criteria for considering a response off-target.

Comparing the Fast-Fourier Transform (FFT) heatmaps, we see that in the responses yielded using factory settings there is an increase in the mid-to-low frequency spectrum (ranging ∼20–10 Hz; note orange- and red-colored bands) and the appearance of high frequencies as well (∼35 Hz; note green bands) compared to the responses obtained with our custom settings (Fig. 2A). Whereas with our custom settings we obtained a tighter color-correlation to the trace, without the appearance of undesired frequencies (note dark blue, with green bands related to the appearance of the traces) (Fig. 2B).

We hereby confirm the differences between traces are obtained as the sum of all the changes in the parameters, not solely as one of the changes alone. All the above indicate that the parameters to be used for detection of the peak responses need to be carefully configured, otherwise the risk of obtaining unwanted artifacts or high signal-to-noise ratio increases considerably.
